# Evaluation of the recovery outcome of poststroke cognitive impairment after cluster needling of scalp acupuncture therapy based on functional near‐infrared spectroscopy

**DOI:** 10.1002/brb3.1731

**Published:** 2020-07-08

**Authors:** Jing Chen, Hui Li, Chao Zeng, Jingge Li, Bin Zhao

**Affiliations:** ^1^ The Third Affiliated Hospital of Zhejiang Chinese Medical University Hangzhou China; ^2^ Heilongjiang University of Traditional Chinese Medicine Harbin China

**Keywords:** acupuncture, cognitive, spectrometry, stroke

## Abstract

**Background:**

Cognitive impairment often arises in patients suffered from stroke. Acupuncture is a recommended treatment option for stroke by the World Health Organization (WHO) and has been shown to improve the cognitive function of patients with poststroke cognitive impairment (PSCI).

**Methods:**

In the present study, we assessed the efficacy of scalp acupuncture with cluster needling on PSCI patients. Fifty six PSCI patients were randomly separated into the reference group who received drug treatment only and the treatment group who received cluster needling of scalp acupuncture on top of drug treatment. Cognitive function was compared between the two groups before and after treatment. We also took the advantage of functional near‐infrared spectroscopy to assess the cerebral hemoglobin levels.

**Results:**

We reveal that applying cluster needling of scalp acupuncture on top of drug treatment can significantly improve the cognitive function and elevate the cerebral hemoglobin levels compared to patients treated with drug only.

**Conclusions:**

Our results suggest that cluster needling of scalp acupuncture is an effective treatment against PSCI and shed light on its application on other neurological disorders.

## Graphical abstract

In the present study, we reveal that applying cluster needling of scalp acupuncture on top of drug treatment can significantly improve the cognitive function and elevate the cerebral hemoglobin levels compared to patients treated with drug only. Our results suggest that cluster needling of scalp acupuncture is an effective treatment against PSCI and shed light on its application on other neurological disorders.

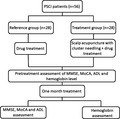

## 1 BACKGROUND

Cognitive impairment is a common complication after a stroke. Approximately 30% of stroke patients develop different degrees of cognitive impairment within three to six months of stroke onset (Cullen, O’Neill, Evans, Coen, & Lawlor, 2007). It is known as a strong predictor for functional recovery in patients with stroke, which not only affects patient's personal life, but also adds a serious burden to the family and society. Poststroke cognitive impairment (PSCI) is defined as the cognitive impairment that arises within six months of stroke onset, including subcortical ischemic infarction, cerebral hemorrhage, and brain degenerative diseases, such as Alzheimer's disease. Incidence of PSCI is related to factors, such as age, education level, stroke type, lesion characteristics, and recurrence. Possible mechanisms include cerebral neurological damage, cerebral small vessel disease, inflammation, neurotransmitter secretion defects, and imbalance in the generation and clearance of amyloid β.

Stroke is among the 43 diseases that are recommended by the WHO for acupuncture treatment. Plenty of clinical evidence in Chinese medicine has suggested that acupuncture can improve the cognitive function of patients with stroke (Ma, 2017). Scalp cluster needling is an acupuncture technique developed by Professor Yu Zhishun, based on his extensive clinical experience in treating cerebral infarction. It is widely used in poststroke recovery and has achieved decent curative effects in swallowing disorders, dyskinesias, and cognitive disorders (Zhang, Jiang, Zhou, & Tang, 2018; Zhu, Chen, Jing, & Fan, 2019). It can effectively improve the Montreal Cognitive Assessment (MoCA) score of patients with mild vascular cognitive impairment. It can also dramatically reduce the latency of P300 potential while increase its amplitude, as well as significantly decrease the homocysteine levels in PSCI patients. Therefore, cluster needling of scalp acupuncture can effectively improve the cognitive dysfunction of stroke patients, especially on the ability of naming, language, and orientation. Cluster needling of scalp acupuncture divides the scalp acupoints into seven regions, each responsible for a specific dysfunction. Application of cluster needling in each region expands the treatment area, increases the amount of stimulation, and enhances the treatment efficacy (Wang, Xu, Zhao, & Fan, 2019). Cluster needling of scalp acupuncture creates a “needle field” for nerve signal input into the corresponding cortex, which improves the excitability of cerebral cortical nervous cells and the hypoxic hyperpolarization of the ischemic penumbra of local neurons, as well as restores the function of nerve fibers.

Poststroke cognitive impairment scoring can be divided into subjective and objective evaluations. Subjective evaluation includes Mini‐Mental State Examination (MMSE) and MoCA (Petersen, 2011). These subjective evaluation methods are simple to perform; however, the test outcome is largely dependent on the patients’ education level and often a single test cannot fully reflect the actual extent of cognitive impairment. Subjective evaluation methods mainly involve the evaluation of physiological and behavioral parameters at the population level. Functional magnetic resonance imaging (fMRI) and positron emission tomography (PET) are imaging methods frequently used to assess the physiological parameters.

Functional near‐infrared spectroscopy (fNIRS) is a newly developed optical brain imaging method, featured by its noninvasive, portable, and low‐cost characters. It can be used for continuous long‐term monitoring of the brain function in real time under a friendly natural condition. It provides a novel option to evaluate cognitive function of the brain. When the near‐infrared light passes through the body, it can be absorbed by hemoglobin to assess the fluctuations in oxygenated and deoxygenated hemoglobin levels. It can reflect small changes in cerebral blood flow during cognitive activities with relatively high temporal resolution. The reliability and authenticity of fNIRS have been verified by fMRI and PET.

In the present study, we applied fNIRS imaging to investigate the therapeutic effect of scalp acupuncture with cluster needling on PSCI.

## 2 METHODS

### 2.1 Patients

Consent was obtained from all participated patients. A total of 56 PSCI patients treated at our institution were included in this study. The patients were randomly divided into a reference group and a treatment group, each with 28 patients. Patients in the reference group received drug treatment only: A daily dose of 5 mg donepezil hydrochloride tablet was taken consecutively for a month. The treatment group received cluster needling scalp acupuncture on top of the drug treatment. A single‐blinded screening method was used to evaluate the recovery status, where the people in charge of the efficacy evaluation were devoid of the grouping information and the people who conduct the data analysis were independent from the specific clinical implementation and design of the project. Stroke was confirmed by head CT and MRI scan. PSCI was diagnosed according to previously published standards (Langa & Levine, 2014).

Patients with the following conditions were excluded from the study. Cognitive impairment was caused by cerebrovascular diseases, such as inflammatory demyelinating diseases of the central system, psychosis, liver and kidney dysfunction, hypothyroidism, and alcoholic encephalopathy. Early manifestations were progressively worsening with memory deficits or other cognitive dysfunctions, such as aphasia, apraxia, and failure to recognize. Patients with severe aphasia or physical dysfunction, severe visual acuity and hearing impairment that affect the examinee with serious primary diseases, such as heart, brain, liver, kidney, and hematopoietic system were also excluded.

### 2.2 Scalp acupuncture with cluster needling

The scalp was divided into seven treatment regions for acupoint selection: parietal and frontal regions responsible for language disorder, temporal region responsible for vision disorder, occipital region responsible for balance disorder, suboccipital region responsible for locomotion disorder, anterior parietal region responsible for swallowing disorder and item region responsible for memory disorder.

Patients were generally set at a seated position, which may be changed under certain conditions. Sterilized 0.25 mm × 40 mm acupuncture needles (ANDE) were used. Needles were pierced 0.5–1 inch deep into the acupoints. The needle was gently penetrated into the skin via a spinning motion at a speed of approximately 200 rounds per minute manually and reached the final position in 10 s. Specifically, the needle was held between the palm surface of the thumb and the radial side of the index finger. Needle rotation was achieved via flexion and extension of the metacarpophalangeal joint of the index finger. A total of 6–8 needles were used for each treatment region, and each treatment course lasted 30 min. After the 30‐min course, all needles were removed, except keeping one long‐lasting needle in each treatment region. The long‐lasting needles were kept at the acupoints for 6 hr to maintain the “needle field” and were rotated every 2 hr. Acupuncture was performed twice a day for 6 days, and a total of four treatment courses were applied to each patient in the treatment group.

### 2.3 Functional near‐infrared spectroscopy

Cerebral blood hemoglobin level was assessed with the ETG‐4000 brain function quantitative imaging device (Hitachi). Each measurement channel consists of a dual‐wavelength LED light source and receiver capable of emitting near‐infrared light at 740 and 850 nm.

The probe arrangement used in this study adopts an international 10/20 system. Through the calibration function of the instrument and the corresponding template, the given channel accurately falls into the prefrontal and parietal lobe movement areas. The template and the head are firmly fixed with an elastic band. When putting the near‐infrared light source probe and the detector probe into the template, in order to ensure that the probe directly contacts the scalp, the subject's hair needs to be fully pulled away. The probe was set to monitor the following cortical regions: left prefrontal cortex, right prefrontal cortex, left motor cortex, and right motor cortex. The template is set as follows: four channels are placed in the frontal lobe, and eight channels are placed in the parietal lobe movement area. To reduce the signal‐to‐noise ratio, the sampling frequency is set to 10 Hz.

### 2.4 Cognitive assessment

Cognitive function was scored by the Montreal Cognitive Assessment (MoCA) (Hu et al., 2013). The MoCA was based on the clinical experience and reference to the mini‐mental state examination (MMSE). It has been widely used to measure tester's awareness and is more sensitive to screening for mild cognitive impairment. The assessing scale involves attention and concentration, executive function, memory, language ability, visual structure ability, abstract thinking ability, computing ability, and orientation. A total score of 26 points or more were considered normal. If the subject has less than 12 years of education, the total score was increased by 1 point. Activity of Daily Living Scale (ADL) is composed of the physical self‐maintenance scale (PSMS) and the instrumental activities of daily living scale (IADL) (Eto et al., 1992) PSMS assesses the following six items: going to the toilet, eating, dressing, grooming, walking, and bathing, with a total of six points. IADL assesses the following eight items: making phone calls, shopping, preparing meals, housework, laundry, using transport to take medicine, and self‐caring, with a total of eight points. The final ADL score was a sum of the score obtained from the PSM and IADL, with a total of 14 points. At least 1 hr was waited between each assessment.

### 2.5 Statistics

All data were entered into the computer and statistically processed using the SPSS 20.0 software. Data were present as mean ± standard deviation. Data that met the normal distribution were compared by independent sample *t* test. Rank data were compared by the rank sum test. Classification data were compared by the chi‐square test. A *p*‐value <.05 indicates statistical difference.

## 3 RESULTS

**Figure 1:**
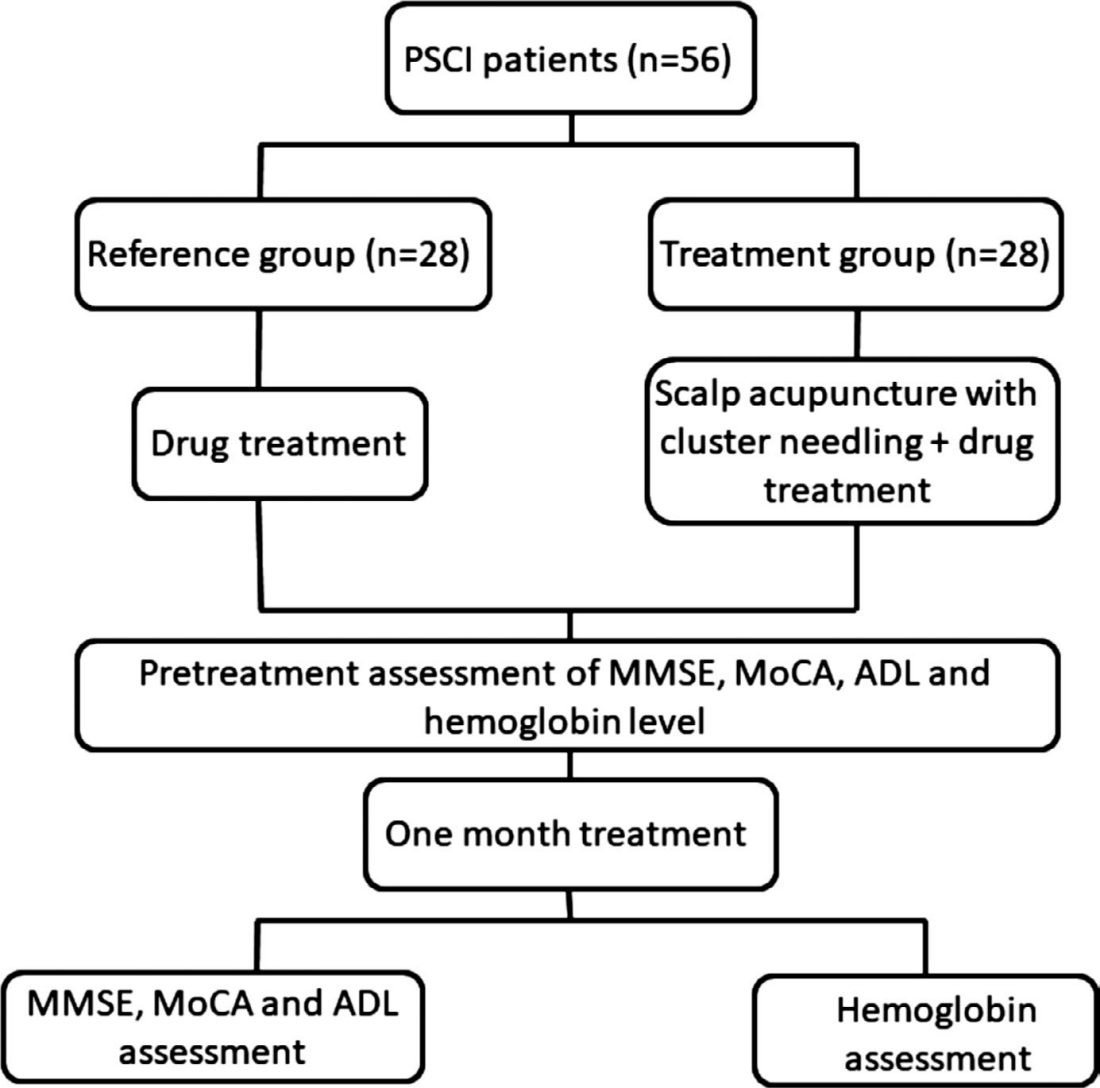
Flowchart illustrating the design of present study

The study was performed according to the flow illustrated in Figure 1. Patient characteristics, MMSE, MoCA, ADL, and hemoglobin levels were assessed right after their diagnosis of PSCI (Table 1). Basic characteristics, including sex, age, body mass index (BMI), and education level, were all comparable between the reference and treatment groups. MoCA and ADL scores were also similar between the two groups, both of which exceeded the standard for regular cognitive function. Cerebral oxygenated (oxy‐Hb), deoxygenated (deoxy‐Hb), and total hemoglobin levels were below the regular standard in all 56 patients, with an average of 9.7 ± 0.8 and 9.8 ± 0.7 g/dl for the two groups, respectively.

**Table 1:** Patient characteristics assessed before treatment

Characteristics	Reference group (*n* = 28)	Treatment group (*n* = 28)	*p*‐value
Sex (male:female)	17:11	16:12	.211
Age	64.5 ± 7.2	65.3 ± 6.8	.322
BMI (kg/m^2^)	23.7 ± 2.6	23.2 ± 2.3	.657
Education level (year)	10.4 ± 3.6	10.7 ± 3.9	.543
MoCA	21.9 ± 2.7	20.7 ± 2.6	.729
ADL	7.0 ± 1.4	6.8 ± 1.5	.482
cerebral oxy‐Hb level (g/dl)	9.1 ± 0.7	9.1 ± 0.6	.913
cerebral deoxy‐Hb level (g/dl)	0.64 ± 0.06	0.62 ± 0.07	.818
cerebral total Hb level (g/dl)	9.7 ± 0.8	9.8 ± 0.7	.897

**Figure 2:**
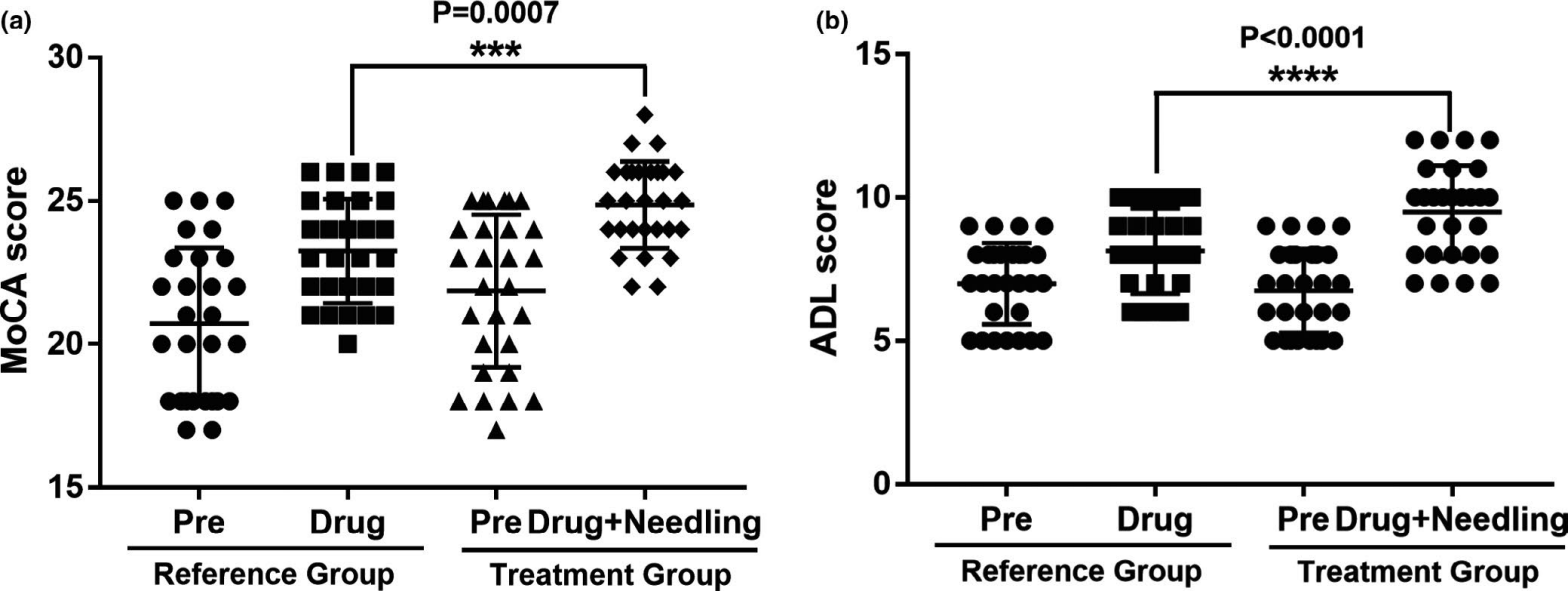
Comparison of MoCA (a) and ADL (b) scores between the reference and treatment groups

After treatment, patients in both the reference and treatment groups exhibited significantly improved MoCA (reference group: 23.3 ± 1.8 vs. 21.9 ± 2.7, *p* = .0262; treatment group: 24.9 ± 1.5 vs. 20.7 ± 2.6, *p* < .0001) and ADL (reference group: 8.1 ± 1.5 vs. 7.0 ± 1.4, *p* = .0047; treatment group: 9.5 ± 1.6 vs. 6.8 ± 1.5, *p* = .0019) scores. More importantly, the improved MoCA and ADL scores were significantly higher in patients from the treatment group compared to the reference group (Figure 2), suggesting that scalp acupuncture with cluster needling on top of drug treatment can further enhance the treatment efficacy on PSCI.

**Figure 3:**
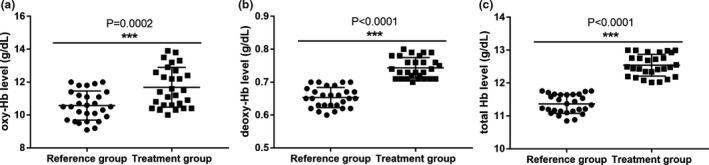
Comparison of cerebral oxygenated hemoglobin (oxy‐Hb) (a), deoxygenated hemoglobin (deoxy‐Hb) (b) and total hemoglobin (c) levels after the corresponding treatments between the reference and treatment groups

For hemoglobin level, the cerebral oxy‐Hb (10.6 ± 0.2 vs. 9.1 ± 0.7, *p* < .0001) and total Hb levels (11.4 ± 0.05 vs. 9.7 ± 0.8, *p* < .0001), but not the deoxy‐Hb level (0.65 ± 0.006 vs. 0.64 ± 0.06, *p* = .3841), were significantly improved in patients treated with drug only in the reference group. Adding cluster needling of scalp acupuncture in addition to the drug treatment not only improved the cerebral oxy‐Hb (11.7 ± 0.2 vs. 9.1 ± 0.6, *p* < .0001) and total Hb (12.5 ± 0.06 vs. 9.8 ± 0.7, *p* < .0001), but also enhanced the deoxy‐Hb level (0.74 ± 0.006 vs. 0.62 ± 0.07, *p* < .0001). Moreover, the extent of cerebral oxy‐Hb, deoxy‐Hb, and total Hb increase was significantly higher in the patients of the treatment group compared to the reference group (Figure 3), suggesting that cluster needling of scalp acupuncture is effective in increasing the cerebral hemoglobin level in PSCI patients.

To explore the correlation between the oxy‐Hb/deoxy‐Hb/total Hb level and the tested neuropsychological scores, we performed Pearson correlation analysis between the three values from patients in both groups and the MoCA and ADL scores. We found that all three Hb levels were positively correlated with the MoCA score and negatively correlated with the ADL score (Tables 2, 3, 4), suggesting that Hb level is positively related to neurological improvement.

**Table 2:** Correlation analysis of oxygenated hemoglobin level with neuropsychological scores

Assessment	Pearson correlation analysis		Partial correlation analysis	
	*r*	*p*‐value	*r*	*p*‐value
MoCA	.213	.009	.327	.011
ADL	−.294	<.001	−.352	<.001

**Table 3:** Correlation analysis of deoxygenated hemoglobin level with neuropsychological scores

Assessment	Pearson correlation analysis		Partial correlation analysis	
	*r*	*p*‐value	*r*	*p*‐value
MoCA	.366	.003	.317	<.001
ADL	−.357	.002	−.211	.002

**Table 4:** Correlation analysis of total hemoglobin level with neuropsychological scores

Assessment	Pearson correlation analysis		Partial correlation analysis	
	*r*	*p*‐value	*r*	*p*‐value
MoCA	.195	<.001	.244	<.001
ADL	−.313	.002	−.354	<.001

## 4 DISCUSSION

Recently, acupuncture has become a regular clinical treatment strategy for cognitive impairment (Wang et al., 2016; Yu, Zhang, Liu, Meng, & Han, 2006; Zhou, Peng, Xu, Li, & Liu, 2015). Neuroimaging has been carried out to investigate the underlying mechanism of acupuncture (Arai et al., 2006; Feng et al., 2012; Wang et al., 2012). In the present study, we investigated the effect of cluster needling of scalp acupuncture on cognitive function recovery and cerebral hemoglobin level in PSCI patients, where the latter was assessed by fNIRS.

fNIRS is a product that detects fluctuations in hemoglobin concentration in the cerebral cortex, characterized by its noninjection, nonradiation damage noninvasive, real‐time spatial resolution, and continuous properties. It employs the near‐infrared light to penetrate the cerebral cortex and reflect back to the receiving probe. Changes in the amount of light absorption reflect the cerebral hemoglobin concentration as an indirect measurement of neural activity in the cerebral cortex. Simultaneous detection of changes in different brain regions is achieved by multi‐channel assessment. At present, fNIRS is mainly used in basic research for movement, sensations (auditory, visual and olfactory), language, cognition, memory, intelligence, emotion, character, decision‐making, sleep, consciousness, gender, development, aging and acupuncture, related research, especially in the field of cognitive neuroscience. In clinical research, fNIRS is mainly used in neurological‐related diseases, various addictive diseases, psychiatric diseases, drug applications, neurological recovery after stroke and injury, pain and epilepsy. It is worth noting that fNIRS can explore the impact of various diagnosis and treatment schemes on the central nervous system of the human body as a whole system, which is in line with the concept of Chinese medicine of viewing the body as an organic unified system. Therefore, it has unique advantages in the research of Chinese medicine‐related diseases and has broad applications in revealing the internal mechanism underlying traditional Chinese medicine treatment.

Given that fNIRS does not involve any brain injury during analysis, it can directly obtain the information of the activation status of different brain functional areas during acupuncture, which enables the in‐depth research on the mechanism of acupuncture. So far, fNIRS‐based research on acupuncture mainly covers difference between central acupuncture during real and fake acupuncture, difference between central acupuncture response by different acupuncture techniques, difference between central acuity response at different acupoints, difference between healthy volunteers and patients with different diseases, and difference between the neural response of thorn stimulation and other types of stimulation. The exertion of acupuncture is closely related to its regulation mechanism on the central nervous system, while there are still different opinions on the particular subject. Chinese medicine acupuncture research team at the Harvard Martinos Biomedical Image Research Center shows that acupuncture works through negative activation of the limbic system, para limbic system, and cerebral cortex. Other studies suggest that the difference in spatiotemporal characteristics of different acupuncture points is the key influencing factors of the treatment efficacy. Therefore, further study is still needed to clarify the mechanism of action underlying acupuncture treatment. In addition, fNIRS has gradually shown great potential application value in other areas of Chinese medicine. For example, fNIRS has been progressively implemented in research on the effect observation, evaluation, and mechanism of traditional Chinese medicine treatment. In addition, other studies are exploring the impact of fNIRS on traditional Chinese medicine symptoms and syndromes.

In this study, the participated patients were separated into the reference and treatment groups by randomizing the admission order of the patients. Cognitive function was assessed with the single‐blind method. People in charge of efficacy evaluation did not know the grouping information of the patients, and people who conducted the data analysis were arranged separately and would not participate in the specific clinical implementation and design of the project. However, the limited sample size is the major drawback of the present study. Data from more patients are needed from future studies to confirm our findings.

## 5 CONCLUSIONS

In summary, we show that application of cluster needling of scalp acupuncture on top of drug treatment can significantly improve the cognitive function and cerebral hemoglobin levels in PSCI patients compared to patients treated with drug alone. Our results provide an indication on the application of cluster needling in combination with fNIRS in the treatment of cognitive impairment.

## CONFLICT OF INTEREST

The authors declare that they have no competing interests.

## AUTHORS' CONTRIBUTIONS

JC, HL, CZ, JL performed the analysis. BZ designed the study and wrote the manuscript.

## Ethical approval

The study has been approved by the ethical committee of the Second Hospital Affiliated Heilongjiang University of Chinese Medicine.

Consent to participate: Consent has been obtained from all participated patients.

### Peer Review

The peer review history for this article is available at https://publons.com/publon/10.10.1111/brb3.1731.

## Data Availability

The data that support the findings of this study are available from the corresponding author upon reasonable request.
